# The Perception of African Immigrant Women Living in Spain Regarding the Persistence of FGM

**DOI:** 10.3390/ijerph182413341

**Published:** 2021-12-18

**Authors:** Ousmane Berthe-Kone, María Isabel Ventura-Miranda, Sara María López-Saro, Jessica García-González, José Granero-Molina, María del Mar Jiménez-Lasserrotte, Cayetano Fernández-Sola

**Affiliations:** 1 Surgical Critical Resuscitation, Torrecárdenas University Hospital, 04009 Almeria, Spain; beretebereteberete@gmail.com; 2 Department of Nursing, Physiotherapy and Medicine, University of Almeria, 04120 Almeria, Spain; jgg145@ual.es (J.G.-G.); jgranero@ual.es (J.G.-M.); mjl095@ual.es (M.d.M.J.-L.); cfernan@ual.es (C.F.-S.); 3 General Surgery Service, La Inmaculada Hospital, 04600 Huercal-Overa, Spain; slopezsaro@gmail.com; 4 Facultad de Ciencias de la Salud, Universidad Autónoma de Chile, Santiago 7500000, Chile

**Keywords:** female circumcision, public health, qualitative research, violence, women

## Abstract

Approximately 200 million women and girls have undergone female genital mutilation (FGM) worldwide. Migration has spread the practice of FGM around the world, thus making it a global public health issue. The objective of this descriptive qualitative study was to explore the perceptions of Sub-Saharan immigrant women in Spain in relation to the causes of the persistence of FGM. In-depth interviews were carried out with 13 female FGM survivors of African origin, followed by inductive data analysis using ATLAS.ti software. Two main themes emerged from the analysis: (1) A family ritual symbolic of purification and (2) a system of false beliefs and deception in favour of FGM. The FGM survivors living in Europe are aware that FGM is a practice that violates human rights yet persists due to a system of false beliefs rooted in family traditions and deception that hides the reality of FGM from young girls or forces them to undergo the practice. The ritualistic nature of FGM and the threat of social exclusion faced by women who have not had it performed on them contributes to its persistence nowadays.

## 1. Introduction

Approximately 200 million women and girls have undergone female genital mutilation (FGM) worldwide and every year, and three million girls are at risk of having this practice performed on them [1,2,3]. FGM is an ancestral practice supported by a complex symbolic system [4,5] that persists in more than 30 countries, being most prevalent in Sub-Saharan Africa and parts of the Middle East and Asia [3]. FGM is also found in areas in Europe, Australia and North America, where, over the last few decades, migrants have travelled from countries in which FGM is practised [3]. According to the European Parliament [6], there are approximately 500,000 women and children in Europe who have undergone FGM and another 180,000 at risk, although these figures may be underestimated, as they do not include undocumented immigrants [7]. In Spain, it is estimated that between 9–15% of girls between the age of 0–18 are at risk of FGM [8]. 

FGM refers to all procedures that involve the partial or total removal of external female genitalia or other injury to the female genital organs for non-medical reasons [9]. According to the WHO, FGM is classified into four types: (I) clitoridectomy, the removal of the clitoris, (II) excision, the removal of the clitoris and the inner labia, (III) infibulation, the narrowing of the vaginal opening by creating a covering seal by cutting and repositioning the inner or outer labia, sometimes sewing them together; and (4) perforation, which has varying degrees of severity within each type of mutilation [1,9]. Some African societies believe that an uncut woman is impure and must therefore be mutilated as proof of her virginity and purity [4,10]. This ritualistic practice has been internationally recognised as an act of violence against the human rights of women and girls [7,11]. Moreover, FGM is a type of discrimination and inequality that this female collective face [12,13]. The prevention/eradication of practices like FGM is the responsibility of the country of origin and the destination country [12,14]. Family and religious communities play an important role in the decision-making process regarding the eradication of FGM [15]. The belief that FGM forms part of one’s cultural identity and is a religious requirement, makes parents and religious leaders pivotal in preventing the problem [16]. The link between social pressure and FGM implicates future generations, who will be the leaders of policies to empower women [17,18].

FGM is not an isolated traumatic event in the life of women who have undergone it [19], but rather the start of life-long physical, obstetrical, sexual and psychological problems [1,20,21,22]. Among the major short-term physical consequences are haemorrhage, infection and shock [23]. In the long term, women present with clitoral neuroma [22], epidermal cysts [24], fistulas [25], bladder dysfunction [21,26], sexual dysfunction, pelvic pain and obstetrical trauma [27]. FGM leads to a higher risk of prenatal mortality both for the mother and foetus [4,27]. Among the psychological consequences, one must highlight severe mental health [4] and psychosexual [23] problems, post-traumatic stress, somatization and low self-esteem [28]. There is a need for research in different countries, cultures, beliefs and organisations regarding knowledge, awareness, prevention and experiences [29]. In Spain, one should address foreign women victims of FGM with a strategy that integrates the physical and psychosexual [30]. Moreover, one should incorporate the perspectives of women, girls, families, healthcare professionals, religious/community leaders and governments [13,28]. Although there is qualitative research that addresses the perspective of healthcare professionals and women in their country of origin [31], little is known about this practice from the perspective of women who have emigrated to Spain [17]. Addressing the problem of FGM in Europe and Spain requires listening to and involving communities from countries of origin where the practice persists [32]. Not only is FGM a problem in the Southern Hemisphere, but it is also a global issue, as migratory movements spread the social structures that provide a breeding ground for this tradition around the world. Furthermore, the reciprocal links and dynamics of factors that support or prevent FGM indicate that the tradition is in transition within practising communities that now live in Western countries [33]. The objective of this study was to explore the perception of Sub-Saharan immigrant women living in Spain, in relation to the causes of the persistence of female genital mutilation. 

## 2. Materials and Methods

### 2.1. Design

A qualitative descriptive study was designed that provides a deep understanding of a particular phenomenon of interest. In the design of descriptive studies, the researcher must integrate their own professional discipline into their work [34]. To report this study, we adhered to the Consolidated Criteria for Reporting Qualitative Research (COREQ), a comprehensive checklist that includes all of the standard criteria for correctly reporting a qualitative study. The criteria included in the checklist can help researchers to report on important aspects of the research team, study methods, the study’s context, results, analysis or interpretations [35]. Even though the COREQ checklist has been subject to criticism [36], it is still recommended for qualitative studies in healthcare [37,38].

### 2.2. Participants and Setting

Thirteen Sub-Saharan women living in the southeast of Spain participated in the study. Convenience sampling was used following these inclusion criteria: to be a female FGM survivor, to have emigrated to Europe and to be an adult (aged 18 and over in Spain). The exclusion criteria were: to refuse to participate, to have cognitive impairment that prevents one from recalling or maintaining a conversation, to not speak Spanish, French, English, Dioula, Bambara or Malenké (African languages spoken by one of the interviewers). To recruit the participants, a researcher contacted professionals, nurses, doctors or midwives from services that usually tend to female FGM survivors (gynaecology, labour ward). These professionals provided the two researchers in charge of carrying out the interviews (midwife and nurse) with data regarding female FGM survivors who received care (pregnancies or gynaecological complications). The interviewers then contacted the women, either by phone or personally when they used a medical service (for example, in the labour ward or gynaecological examinations). Of the 18 women affected, five women refused to participate, of which two stated that they did not want to recall their experience and three due to potential family reprisals. The sociodemographic data of the participants can be found in Table 1.

### 2.3. Data Collection

Data collection included in-depth interviews carried out between January 2020 and June 2021 in an office at the University of Almería, a community healthcare centre in Almería and three private homes. Two researchers who received training in qualitative research (Research Masters), and one of whom spoke various African dialects carried out the interviews. Before starting the interview, the interviewers practised the in-depth interview protocol (Table 2). Additionally, the participants were informed about the objective and the ethical issues of the study (confidentiality, voluntary nature of participation, permission to record), and they gave informed consent verbally and in a signed document. After this, the first question was asked to start the dialogue (see the question script and interview protocol, Table 2). The interviews lasted on average 86 min and were recorded to facilitate their subsequent transcription. The interviewers took notes on non-verbal and paraverbal aspects of communication (gestures, tone of voice…). When the interviewers perceived that no new information was being provided, they considered that data saturation had been reached [39] and ended data collection.

### 2.4. Data Analysis

The transcriptions were incorporated into an ATLAS.ti 9 project, along with the researchers’ notes. The thematic analysis used an inductive strategy following the phases described by Braun and Clarcke [40]. Phase 1. Data familiarization, which consisted of a full reading of all of the transcriptions in order to extract general meaning and then a re-reading in which familiarization notes were written using ATLAS.ti’s memo function. Phase 2. Systematic data coding, in which the transcriptions were given codes using various procedures of the ATLAS.ti software (initial phase, open coding, live coding, when codes were reused, coding by list…) Phase 3. Generating initial themes from codes and data collected: The codes were well established rather than anecdotal and were grouped thematically in themes that have shared meaning patterns, connected by a central concept or idea. Phase 4. Development and revision of the themes, ensuring that the developed themes were consistent with the codes that grouped them and with the coded quotations. In this phase, ATLAS.ti was used to generate networks that represent a conceptual map of the analysis. Phase 5. Refining, defining and naming themes. Phase 6. Writing the report, during which the most explanatory examples (quotations) were selected, and summaries of eloquent examples were written. The analysis was linked again to the research question and the literature. Three researchers (OBK, SMLS, CFS) carried out the coding process. The codes that did not achieve 66% consensus were removed or modified (phase 2). The themes were provisionally named by three encoders (phase 3) and were later refined and validated by the whole team (phases 5 and 6).

### 2.5. Rigor

Credibility: the participants and researchers familiarised themselves with the context of the study to guarantee trust and valuable data. Various researchers participated in the coding process, analysis and interpretation of the data. Transferability: the study setting, the participants, the context and the method were described in detail. Reliability and confirmability: the researchers did the transcriptions and then other members of the research group revised and verified them. The researchers examined their own values and preconceptions regarding the topic in question before describing the results.

### 2.6. Ethical Considerations

This study was approved by the Ethics Committee of the University of Almería’s Nursing, Physiotherapy and Medicine Department (Protocol number: EFM-03/20). The participants were informed of the objective/aim of the study and the voluntary nature of participation. Permission was asked to be able to record the conversations and informed consent was signed. Confidentiality and anonymity were guaranteed by giving codes to the participants’ names. In all phases of the study, the current ethical principles of the Declaration of Helsinki were taken into account.

## 3. Results

**Figure 1:**
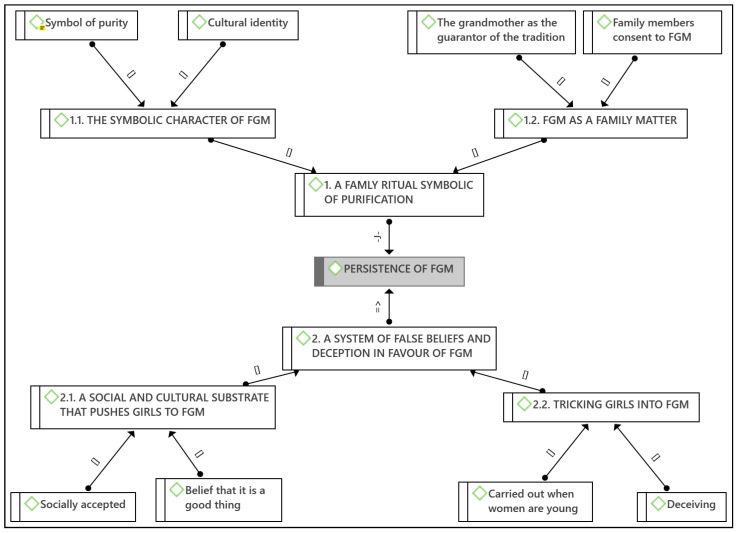
Conceptual map of themes and emerging codes. Key: [] is par of; => is because of; -J- justifies.

Thirteen female FGM survivors took part in the study with an age range of 26–35 years (average: 29.62, SD: 3.01) (See Table 1). Two main themes emerged from the analysis, which helped us to explore the causes of the persistence of FGM from the perspective of its survivors (Figure 1).

### 3.1. A Family Ritual Symbolic of Purification

All of the participants highlight the widespread social acceptance of FGM in the communities in which it is practised due to the belief that the practice has been inherited from its ancestors and that it allows a woman to be purified. Two subthemes arose from this theme: 

#### 3.1.1. The Symbolic Character of Female Genital Mutilation

FGM is an ancestral cultural practice that persists in many ethnic groups, reflecting the importance of traditional customs that are difficult to eradicate.

*“Cutting is tradition. Every woman in my country is cut, even the president’s wife… all of them. It is our tradition and I want to respect it but it has brought me no good whatsoever”*

(P7)

*“Girls are mutilated at a young age in our culture…It is a long tradition, hundreds of years old, they don’t know that they have been cut…”*

(P8)

The participants also describe a wide diversity of practices and rituals that form part of the process. Some stated that FGM is understood in their society and culture as the rite of passage from childhood to the first phase of adulthood. It is also considered their evolution towards sexual maturity and reproductive age as it coincides with their first menstruation.

*“I was 8 or 9 when they did it. I have vague memories of it. Everyone was happy about it because they said that I had become a woman”*

(P4)

The participants associate the persistence of FGM with social values such as a woman’s virginity and purity.

*“For the last 3000 years families have believed that a girl who has not been cut is impure because what they have between their legs is impure so it must therefore be closed as proof of virginity and purity”*

(P1)

The participants perceive that girls who have been mutilated are more appreciated my male members of the community, as this guarantees fidelity and control over the woman in sexual terms. Indeed, it can be considered a patriarchal practice in which a man controls a woman’s sexuality.

*“Those who don’t have it are frowned upon. She is a woman who has relations with various men. It is done so that women maintain their virginity until marriage and so that they don’t like or want to have sexual relations.”*

(P5)

*“They say that when you cut a woman, she doesn’t want to have sex, she’s not going to look for it, until her husband wants it.”*

(P11)

Some participants allude to the cultural traditions of their countries of origin, which deem women impure from birth, thus needing to be mutilated in order to be purified and develop their sexuality.

*“My Dad is Muslim and he says that cutting is a good thing, the problem is the woman. My husband says that it is tradition, he doesn’t want uncut and impure woman.”*

(P4)

The women who have not undergone the practice are considered impure, unfaithful, polygamous and are generally stigmatized or rejected in some societies. There are even men who refuse to marry them:

*“When a woman has not been cut, they don’t consider her one of theirs. I’m not sure how to put it, for them it’s that she does not have the same value.”*

(P10)

#### 3.1.2. Female Genital Mutilation as a Family Matter

In these cases, the ritualistic nature is evident both in the preparation (the party, singing women, drums) as well as in the celebration after the cut. This is how one participant describes it:

*“They got us dressed in white and we had to walk around the whole village, my grandmother’s village, and they took us around the neighbourhood showing everyone that we had undergone the practice, like they were celebrating it.”*

(P1)

The social setting aims to associate FGM with feelings of family belonging. Not undergoing FGM would lead to being excluded from their community, to being different or to not identifying with their culture. Thus, the practice is not carried out with negative intentions or with the aim of causing damage, but rather because it is considered positive. One participant describes how not undergoing FGM can lead to social exclusion.

*“When a girl has not been cut, she is not considered one of them, (…) she does not have the same worth.”*

(P10)

FGM is sustained by a family support system in which the parents accept it or directly promote it, and the grandmothers become the guarantors of the tradition. In a society in which family is sacred, this is an important factor in the persistence of FGM.

*“… I got really angry with my mother. She explained to me that in Africa women are subjected by their families. Family is incredibly important and a woman is subservient to her husband and the grandmothers who want to continue with the tradition.”*

(P11)

*“My mother should have said no to my grandmother because she is my mother and the person responsible for me, but I suppose I understand…, she couldn’t really do anything about it.”*

(P6)

### 3.2. A System of False Beliefs and Deception in Favour of FGM

The system surrounding FGM is made up of a series of false beliefs and deception towards girls, which contributes to the persistence of this practice. Two subthemes arose from this theme:

#### 3.2.1. A Social and Cultural Substrate That Pushes Girls to Female Genital Mutilation

Some participants hold the false belief that FGM is linked to the Bible or the Koran, but others deny this association with a particular religion. The practice is carried out by Muslims, Christians, Jews and Animists. It is associated with the woman’s need for chastity, virginity, purity and cleanliness. Despite the beliefs of some of the participants, the practice is not reflected in any religious text.

*“It is a tradition. Before it was done on all women because it is in the Bible and the Koran. It is a tradition, when a girl is born, she has to be cut. It’s normal, normal traditional things, the elder ladies of the village perform it.”*

(P2)

#### 3.2.2. Tricking Girls into Female Genital Mutilation

However, it is not a rite of passage in all ethnic groups, as in some of them the practice takes places at an age that is not at all related to a particular transitory phase. In some ethnic groups, FGM is a systematic practice that affects very young girls that do not have an alternative. Many girls do not remember having undergone the process.

*“They did it to me when I was a baby, when I was a few months old.”*

(P4)

*“Before, when a girl was born, they had to commit to the tradition straight away and apply it to everyone.”*

(P10)

Some of the participants stated that girls are often deceived by telling them that they are going to a birthday or other party. The festive nature of the tradition, including music, drums, special meals and a breakfast feast, can lead the girls to feel happy yet scared at the same time.

*“My Mum got me dressed up and told me we were going to a birthday party.”*

(P6)

*“Everything started one day in my grandmother’s house. I remember that… she fooled us with a special breakfast… I can’t remember what kind of breakfast it was, but it was really good.”*

(P1)

*“In that moment I didn’t understand and I was almost frightened, but happy, I was only a young girl who had no idea about all of this. Everyone was happy and celebrating.”*

(P1)

In other cases, the girls simply had the truth kept from them, not knowing where they were going the day that they were mutilated. They were told they were just going to see their grandmother or some other pretext. Once they were there, they covered their eyes so that they could not see what was happening to them:

*“My Mum told me that she had to go to my grandmother’s house to do something. I saw a woman with a knife but I don’t remember much else, they just covered my eyes.”*

(P3)

The deception is not just a way of leading girls to undergo FGM, but also as a means to hide what has happened from them. Some women stated that, as girls, they were not aware that they had been subjected to FGM until they discovered it talking with their friends:

*“A few days afterwards, some friends came over to play and they told me that I had been cut, how do you say… that they had cut my clitoris. That’s when I found out because I hadn’t understood what had happened.”*

(P6)

## 4. Discussion

The objective of this study was to explore the perceptions of Sub-Saharan immigrant women living in Spain in relation to the causes of the persistence of FGM. According to the participants’ accounts, FGM is a cultural practice, and social acceptance is so important to them that they accept the procedure even if it has had many negative consequences. 

This practice is considered a rite of passage from childhood to the first phase of adulthood, meaning that it should be carried out closer to puberty than childhood [41]. The participants who underwent the procedure closer to puberty remember it as a celebration. However, in other ethnic groups, it is done as early as possible and with no alternative, as it is not considered a rite of passage, thus meaning that those who were subjected to it as babies do not remember anything.

The strong sense of family belonging surrounding this practice is another justification for the practice, in which grandmothers are the ones who carry it out. Even if the mothers do not agree with the practice, they eventually accept it. The WHO consider that this traditional practice has negative consequences on female sexual function and basic rights [4,30]. The WHO, as well as other international organisations, regard FGM as one of the worst forms of violence, not only because it is practised on young and defenceless girls, but also due to its highly negative emotional, physical and psychosocial ramifications [25,42,43]. 

FGM is a culturally accepted form of torture according to some researchers [4,21], and other studies [10,44,45] regard FGM as a way of humiliating women and girls. In fact, the perception surrounding the consequences of FGM has a direct impact on our participants’ stance regarding the practice. 

Additionally, it is important to disassociate the practice with religious beliefs, as FGM does not figure in any religious text [14,16], yet it is considered a justification by some of our participants. FGM should also be deemed a cultural issue, not a religious one, as there are many strongly religious countries such as Saudi Arabia and Morocco, in which it is not practised [16,45]. Removing any religious connotation from FGM amongst the potential risk groups would help reduce popular support of the practice. The groups who carry out FGM are unaware of its negative effects on the health and wellbeing of women, which is why it is of vital importance that healthcare professionals make these consequences known [12,16,46]. 

Another reason for justifying this practice is psychosexual. As our participants state, girls who have been mutilated are more appreciated by the men in their community, demonstrating that this practice is a way of controlling women’s sexuality. Publishing the negative consequences of FGM in relation to sexuality could be beneficial [16,47,48]. An action protocol must also be developed to facilitate healthcare professionals’ work in preventing the ablation [18,45].

For other authors [49,50], FGM is a painful practice, carried out under non-existent hygiene conditions leading to pain that can compromise one’s life. Furthermore, according to results, this female collective is forced to undergo this practice [3]. Corroborating our results, FGM violates the rights of Sub-Saharan women [10,44].

In line with other studies [17,51,52], there is a consensus regarding the need to eradicate FGM. Ablation is a practice that is considered an assault, an attack on women’s rights, dignity and integrity [53]. However, the measures are limited; the most significant actions in terms of caring for the victims and risk groups have only taken place on a local level. 

In agreement with the participants’ perceptions, Western societies’ healthcare professionals must attend to African immigrant women with individualised and culturally sensitive care [22,53,54,55]. Indeed, migrant women affected by FGM require advice and specialised care [56]. If gender violence is understood without taking into account each individual sociocultural context, policies to eradicate it become an ideological tool that can do even more damage to the women affected by FGM. In this regard, and choosing an approach that considers the intersectionality of gender, ethnicity, culture, religion, class, sexuality and economic status as multiple factors connected to gender inequality, allows for alternatives that protect the health of African girls in Europe without undermining their culture or creating further marginalisation [57].

Previous studies have demonstrated that, in comparison with other European countries, the Spanish system is not well-prepared in detecting potential FGM cases [22,28,53]. Whilst FGM has a prevalence of almost 95% in countries of origin, evidence shows that it is not currently practised in host countries [25,27].

The sample size is an important limitation of the study. That was due to the difficulty in finding FGM survivors willing to participate. Communication barriers are also a typical obstacle to helping and researching people with diverse linguistic and cultural backgrounds [54]. Some of the migrants refused to participate in this study due to either not feeling psychologically prepared, fear of potential family reprisals or respect for tradition. The age range of the participants is very narrow (26–35 years old). This is due to the recent nature of female migration from Sub-Saharan countries [58], thus the lack of older women from those countries. Including older women could have provided different results, as the study suggests that the acceptance of the practice increases with age (grandmothers are the guarantors of the tradition). The participant sample is homogenous in terms of gender, but the sociocultural characteristics differ depending on the country of origin.

## 5. Conclusions

African survivors of FGM living in Spain consider that the persistence of the practice is due to the ancestral nature of a custom that provides identity and social inclusion for women who undergo FGM. In certain communities, FGM is justified due to the importance of social acceptance, the family’s role, in particular that of the grandmothers, and the purifying ritualistic nature of the practice. 

FGM persists due to a system of false beliefs and deception aimed at young girls who undergo the practice. To ensure the persistence of the practice in certain ethnic groups or cultures, it is tied to religion, despite not appearing in any religious text of the major world religions. Furthermore, girls are deceived or have the procedure carried out on them at a very young age so that they do not remember it. It is a practice that persists unofficially in order to exercise control over women’s sexuality. 

Implications. Healthcare professionals must make migrant women at risk of suffering FGM aware of it. In order to address the issue, it is important to be respectful in order to avoid cultural conflict. A multidisciplinary team in Spain or in other European or global communities with different specialities must be formed in order to develop preventative strategies and awareness regarding FGM with the aim to eradicate the practice and to dispel the false beliefs surrounding FGM. The creation of action protocols should take into account the following: (a) the risk for girls who travel to visit their grandmothers in Africa. In the event of risk, alerts should be activated and a document should be signed stating that the practice is not allowed in Spain, which could result in legal problems upon returning or losing custody of the girls in question; (b) cooperating with healthcare in schools: one should monitor absenteeism (which could be the sign of an uncommunicated trip) and behavioural changes in the girls; (c) collaborate with social workers: create peer groups (facilitate women meeting and talking about their experience/fears); (d) conduct workshops to dispel false beliefs and therefore prevent women from carrying out this practice on their daughters. In this way, making people aware of the severe consequences of this practice could be a key factor in eradicating it. Lastly, more research is required to address the psychological impact of FGM on women in more detail, and to obtain a male perspective on the issue, given that many women mention male figures in relation to the practice.

## Figures and Tables

**Table 1:** Sociodemographic characteristics of the participants.

Participant	Gender	Age	Religion	Country of Origin	Age FGM
P1	Female	35	Christian	Gambia	7
P2	Female	32	Muslim	Senegal	months
P3	Female	26	Christian	Equatorial Guinea	4
P4	Female	30	Muslim	Guinea Conakri	6
P5	Female	28	Muslim	Gambia	3
P6	Female	29	Christian	Nigeria	5
P7	Female	32	Muslim	Burkina Faso	1
P8	Female	34	Muslim	Mali	3
P9	Female	26	Muslim	Senegal	6
P10	Female	30	Muslim	Mali	2
P11	Female	27	Muslim	Burkina Faso	4
P12	Female	26	Muslim	Senegal	4
P13	Female	30	Muslim	Mali	months

**Table 2:** Interview protocol.

Stage of the Interview	Topic	Content/Example Questions
Presentation	Aims	Belief that their perceptions of FGM provide an important lesson that must be known.
	Intentions	To carry out research to shed light on these perceptions.
	Ethical aspects	Inform about: voluntary nature, confidentiality, anonymity, possibility to withdraw or not answer, permission to record.
Initial phase	Opening questions	Tell me about yourself and your ethnic origin.
Development	Conversation guide	What does FGM mean to you? What does it mean for your family and community? Why does this practice persist?
Closing	Final questions	Is there anything else that you would like to add?
	Gratitude	Thank you for your time. Your contribution will be very useful to us.
	Offer	We remind you that you can call us should you have any questions. We will inform you about the results of our study.

## Data Availability

For confidentiality purpose, the primary data (interview records) are in possession of the main author (OBK). Transcripts are in a project of ATLAS.ti, and this software is required to access them.
